# Cyclic Adenosine Monophosphate (cAMP)‐Dependent Phosphodiesterase Inhibition Promotes Bone Anabolism Through CD8 ^+^ T Cell Wnt‐10b Production in Mice

**DOI:** 10.1002/jbm4.10636

**Published:** 2022-05-31

**Authors:** Susanne Roser‐Page, Daiana Weiss, Tatyana Vikulina, Mingcan Yu, Roberto Pacifici, M. Neale Weitzmann

**Affiliations:** ^1^ Atlanta Department of Veterans Affairs Medical Center Decatur GA USA; ^2^ Division of Endocrinology and Metabolism and Lipids, Department of Medicine Emory University School of Medicine Atlanta GA USA

**Keywords:** ANABOLICS, THERAPEUTICS, OSTEOBLASTS, CELLS OF BONE, OSTEOIMMUNOLOGY, SYSTEMS BIOLOGY–BONE INTERACTORS

## Abstract

Cyclic adenosine monophosphate (cAMP)‐dependent phosphodiesterase (PDE) inhibitors such as pentoxifylline (PTX) suppress cAMP degradation and promote cAMP‐dependent signal transduction. PDE inhibitors increase bone formation and bone mass in preclinical models and are used clinically to treat psoriatic arthritis by targeting inflammatory mediators including activated T cells. T cell activation requires two signals: antigen‐dependent CD3‐activation, which stimulates cAMP production; and CD28 co‐stimulation, which downregulates cAMP‐signaling, through PDE activation. PDE‐inhibitors consequently suppress T cell activation by disrupting CD28 co‐stimulation. Interestingly, we have reported that when CD8^+^ T cells are activated in the absence of CD28 co‐stimulation, they secrete Wnt‐10b, a bone anabolic Wnt ligand that promotes bone formation. In the present study, we investigated whether the bone anabolic activity of the PDE‐inhibitor PTX, has an immunocentric basis, involving Wnt‐10b production by CD8^+^ T cells. When wild‐type (WT) mice were administered PTX, biochemical markers of both bone resorption and formation were significantly increased, with net bone gain in the axial skeleton, as quantified by micro‐computed tomography (μCT). By contrast, PTX increased only bone resorption in T cell knockout (KO) mice, causing net bone loss. Reconstituting T cell–deficient mice with WT, but not Wnt‐10b knockout (KO) CD8^+^ T cells, rescued bone formation and prevented bone loss. To study the role of cAMP signaling in Wnt‐10b expression, reverse‐transcription polymerase chain reaction (RT‐PCR) and luciferase‐reporter assays were performed using primary T cells. PDE inhibitors intensified Wnt‐10b promoter activity and messenger RNA (mRNA) accumulation in CD3 and CD28 activated CD8^+^ T cells. In contrast, inhibiting the cAMP pathway mediators protein kinase A (PKA) and cAMP response element‐binding protein (CREB), suppressed Wnt‐10b expression by T cells activated in the absence of CD28 co‐stimulation. In conclusion, the data demonstrate a key role for Wnt‐10b production by CD8^+^ T cells in the bone anabolic response to PDE‐inhibitors and reveal competing T cell–independent pro‐resorptive properties of PTX, which dominate under T cell–deficient conditions. Selective targeting of CD8^+^ T cells by PDE inhibitors may be a beneficial approach for promoting bone regeneration in osteoporotic conditions. © 2022 The Authors. *JBMR Plus* published by Wiley Periodicals LLC on behalf of American Society for Bone and Mineral Research.

## Graphical abstract

The phosphodiesterase (PDE) inhibitor pentoxifylline (PTX) promotes bone anabolism in the vertebrae of WT mice by a mechanism involving CD8^+^ T‐cell production of Wnt‐10b.

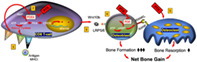

## Introduction

The immunoskeletal interface is a nexus between immune and skeletal systems, resulting from the repurposing of immune cells and their cytokine mediators, for skeletal functions.^(^ ^1^ ^)^ As a consequence, inflammation and other immunological perturbations have the potential to drive bone loss in multiple conditions including rheumatoid arthritis, periodontitis, Crohn's disease, estrogen deficiency, hyperparathyroidism, human immunodeficiency virus (HIV) infection, and immune reconstitution following antiretroviral therapy against HIV.^(^ ^1^, ^2^ ^)^ In these states, bone loss is driven by osteoclastogenic factors including receptor activator of nuclear factor κB ligand (RANKL), tumor necrosis factor α (TNFα), interferon γ (IFNγ), and interleukin (IL)‐17A, secreted by activated adaptive immune cells including CD4^+^ and CD8^+^ T cells, B cells, and macrophages.^(^ ^1^, ^2^, ^3^, ^4^, ^5^, ^6^ ^)^

Interestingly, anti‐osteoclastogenic roles of CD8^+^ T cells have also been recognized, although until recently, the mechanisms of action could not be determined.^(^ ^7^, ^8^ ^)^ Intriguingly, studies now reveal that exposure of CD8^+^ T cells to low‐dose RANKL can induce their differentiation into FoxP3^+^ immunosuppressive CD8^+^ regulatory T cells (Tregs)^(^ ^9^ ^)^ that function to limit bone resorption in ovariectomized mice^(^ ^10^ ^)^ as well as stimulate bone formation.^(^ ^9^ ^)^

In contrast to anti‐cataboliFc activities of CD8^+^ T cells, our group has reported that the bone anabolic activity of parathyroid hormone (PTH) in mice, and of teriparatide in humans^(^ ^11^ ^)^ is dependent in part, on production of the bone anabolic factor Wnt‐10b by CD8^+^ T cells.^(^ ^12^, ^13^, ^14^ ^)^ PTH has further been reported to mediate regenerative effects in human periodontal ligament cells via Wnt‐10b production.^(^ ^15^ ^)^

Abatacept is a pharmacological immunosuppressant, used in the therapy of chronic inflammatory states including rheumatoid arthritis and transplant rejection. Abatacept is based on the physiological CD28 co‐stimulation inhibitor, cytotoxic T‐lymphocyte antigen‐4 (CTLA‐4) a major product of activated T cells and Tregs. CTLA‐4 mediates anti‐resorptive activity, by virtue of its immunosuppressive action, in PTH‐induced bone loss,^(^ ^16^ ^)^ but has also been reported to directly bind to osteoclast precursors in vitro, inhibiting their differentiation.^(^ ^17^ ^)^

Interestingly, we have reported that abatacept is not only anti‐resorptive, but in healthy wild‐type (WT) mice stimulates bone formation and bone accrual, by driving Wnt‐10b secretion by T cells.^(^ ^18^, ^19^ ^)^ This effect is counterbalanced by a negative feedback loop involving sclerostin production following blockade of CD80/86 ligands on osteoblast‐lineage cells.^(^ ^19^ ^)^

We have uncovered a similar paradigm, in which the T cell CD40 ligand inhibitor MR1 induced gain of trabecular bone mass in the spine due to an increase in bone formation, that was associated with increased Treg development and elevated production of CTLA‐4, leading to Wnt‐10b production by T cells.^(^ ^20^ ^)^

At the molecular level, the mechanisms by which Wnt‐10b is generated by CD28 co‐stimulation blockade in T cells is unclear; however, the cAMP signal transduction pathway plays a key regulatory role in the process of T cell activation.^(^ ^21^, ^22^ ^)^ cAMP is generated following engagement of the T cell receptor (TCR) with antigen, presented by antigen presenting cells (APCs). cAMP signaling contributes to driving T cells to anergy, a dormant state, unless the cAMP signal is reversed through co‐stimulation by CD28, a receptor on T cells that is activated by CD80/86 ligands expressed by APC. CD28 signaling activates a phosphodiesterase (PDE) in the T cell that catabolizes cAMP to inactive AMP, thus allowing T cell activation, proliferation, differentiation, and ultimately, downstream effector functions.^(^ ^21^, ^23^, ^24^, ^25^ ^)^

cAMP is an activator of protein kinase A (PKA) that phosphorylates and activates the cAMP response element‐binding protein (CREB), a transcription factor which associates with cAMP response elements (CREs) in the promoters of certain genes, including those involved in T cell activation such as IL‐2.^(^ ^26^ ^)^ Because cAMP has been reported to regulate basal Wnt‐10b expression in adipocytes,^(^ ^27^ ^)^ the premise of this study was that the cAMP/PKA/CREB pathway may also contribute to Wnt‐10b production by CD8^+^ T cells.

PDE inhibitors such as pentoxifylline (PTX) and rolipram, which block cAMP catabolism enhancing intracellular concentrations of cAMP, have long been recognized as stimulators of bone formation^(^ ^28^ ^)^ and have recently come under renewed attention as potential bone regenerative agents.^(^ ^29^, ^30^, ^31^, ^32^ ^)^ However, the mechanisms by which PDE inhibitors induce bone formation have not been clarified, although it has been suggested that they may function by mimicking the downstream actions of PTH on osteoblasts, or by suppressing production of TNFα by macrophages,^(^ ^28^ ^)^ a factor known to suppress bone formation.^(^ ^33^ ^)^ Indeed, recent studies in vitro using monocytes and peripheral blood mononuclear cells from human psoriatic arthritis patients, suggest that apremilast, a US Food and Drug Administration (FDA) approved PDE4 inhibitor, used for treatment of psoriatic arthritis, may inhibit osteoclastogenesis by suppressing production of inflammatory cytokines including TNFα and IL‐17A.^(^ ^34^ ^)^ Other in vitro studies have reported that PTX‐induces vascular endothelial growth factor, which could play a role in promoting bone formation directly.^(^ ^29^ ^)^

In the present study, we investigated the mechanisms underlying the bone anabolic response of the PDE inhibitor PTX in mice in vivo. Our data reveal that PTX induces a state of increased bone turnover with a net increase in bone mass in the axial skeleton, and that CD8^+^ T cells and Wnt‐10b are mandatory for this bone anabolic response. By contrast, PTX‐induced bone resorption was independent of T cells, and was the dominant effect on bone in T cell–deficient mice.

## Materials and Methods

Pentoxifylline and all other reagents were purchased from the Millipore‐Sigma Aldrich Chemical Co. (St. Louis, MO, USA), unless indicated.

### Mice

Animal studies were approved by both the Atlanta VA Medical Center and Emory University Animal Care and Use Committees and were conducted in accordance with the NIH Laboratory Guide for the Care and Use of Laboratory Animals.

Mice were housed under specific pathogen free conditions and fed 5V02 mouse chow (Purina Mills, St. Louis, MO, USA) and water ad libitum. The animal facility was kept at 23 ± 1°C, with 50% relative humidity and a 12/12‐hour light/dark cycle.

**Fig. 1:**
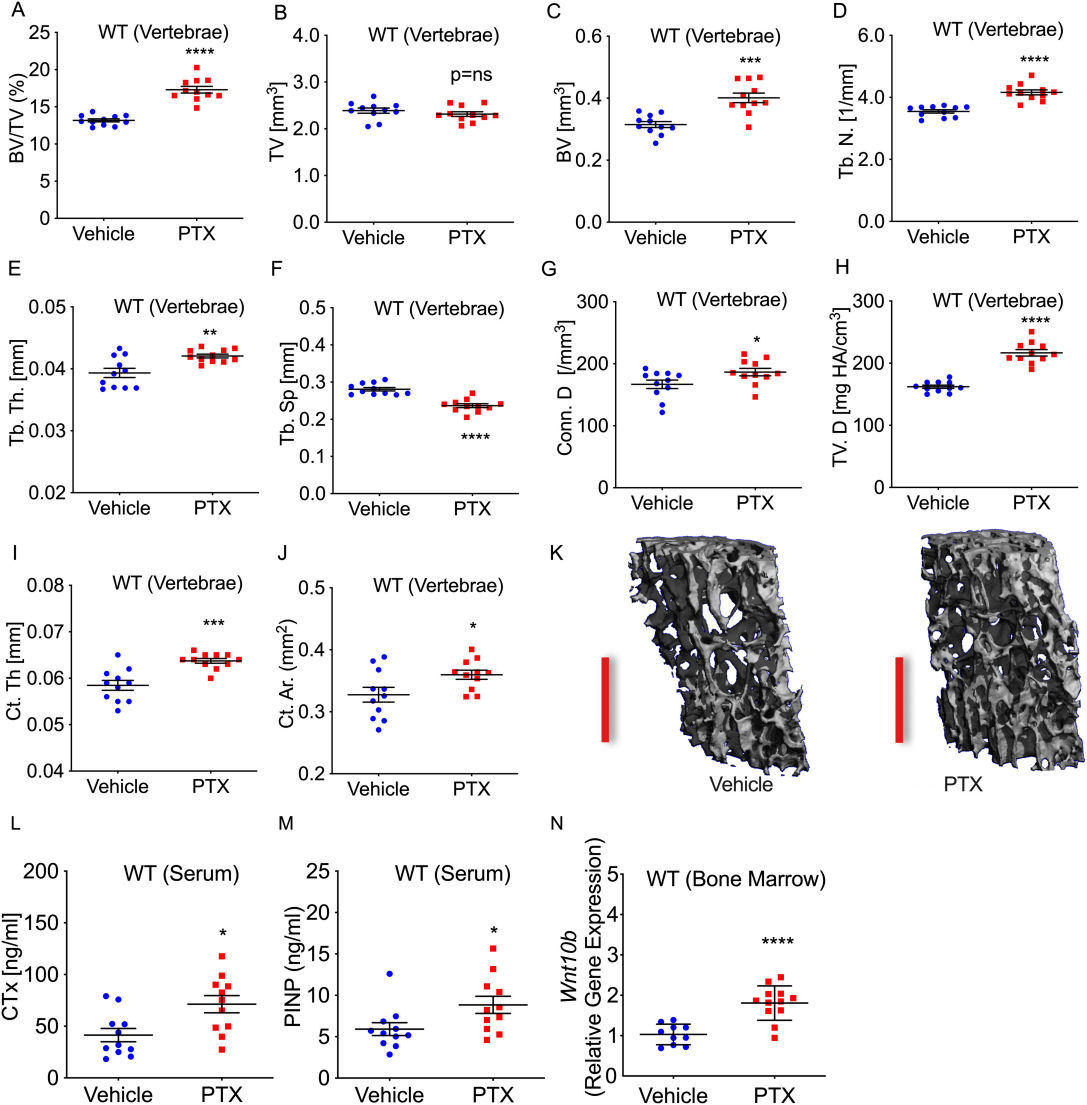
PTX induces both bone formation and resorption in WT mice leading to increased lumbar spine trabecular and cortical bone mass. Two‐month‐old WT C57BL6/J female mice were treated with PTX or vehicle (PBS) 5 times/week, for 12 weeks. Vertebral (L_3_) trabecular and cortical structure was analyzed by μCT and serum bone turnover markers by ELISA. μCT trabecular indices: (*A*) BV/TV; (*B*) TV; (*C*) BV; (*D*) Tb.N; (*E*) Tb.Th; (*F*) Tb.Sp; (*G*) Conn.D; and (*H*) TV.D. μCT cortical indices: (*I*) Ct.Th and (*J*) Ct.Ar. (*K*) Representative 6‐μm μCT reconstructions of vertebrae for vehicle‐treated and PTX‐treated mice. Red scale bar = 500 μm. (*L*) Bone resorption marker CTx and (*M*) bone formation marker P1NP. (*N*) RT‐PCR analysis of *Wnt‐10b* gene expression in bone marrow. Mean ± SEM. *p < 0.05; **p < 0.01, ***p < 0.001, ****p < 0.001, or *p* = ns (not significant) by Student's *t* test or Mann‐Whitney test (P1NP) following assessment for normal distribution by Shapiro‐Wilk normality test. *n* = 11 mice/group. One vehicle RT‐PCR sample was undetectable in *N*. BV = bone volume; BV/TV = trabecular bone volume fraction; Conn.D = connectivity density; Ct.Ar = cortical area; Ct.Th = cortical thickness; CTx = C‐terminal telopeptide of collagen type I; P1NP = N‐terminal propeptide of type‐I procollagen; Tb.N = trabecular number; Tb.Sp = trabecular separation; Tb.Th = trabecular thickness; TV = tissue volume; TV.D = volumetric bone density.

**Fig. 2:**
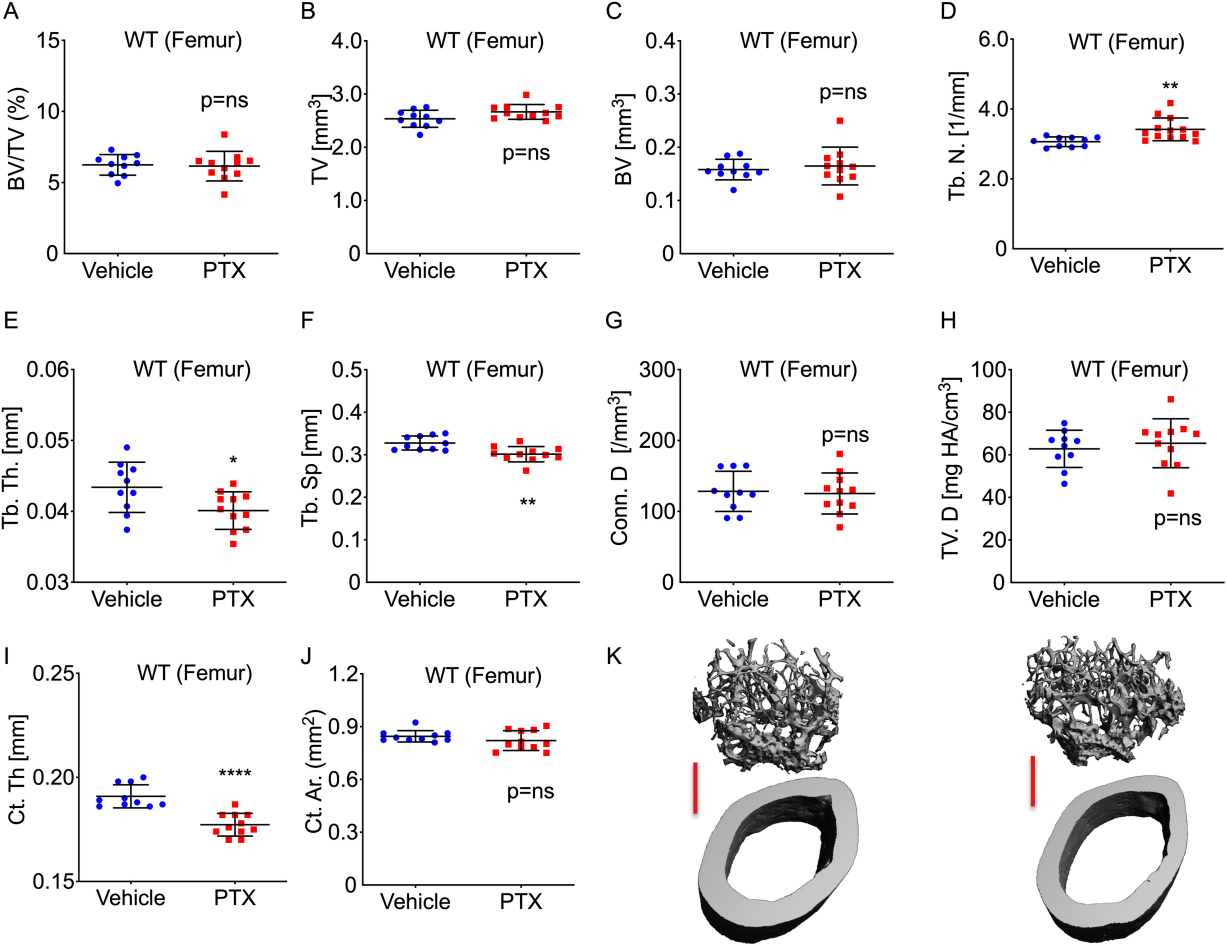
PTX does not increase femoral trabecular and cortical bone mass in WT mice. Two‐month‐old healthy intact WT C57BL6/J female mice were treated with PTX or vehicle (PBS) 5 times/week, for 12 weeks and femoral bone analyzed by μCT. Trabecular bone indices: (*A*) BV/TV; (*B*) TV; (*C*) BV; (*D*) Tb.N; (*E*) Tb.Th; (*F*) Tb.Sp; (*G*) Conn.D; and (*H*) TV.D. Cortical indices: (*I*) Ct.Th and (*J*) Ct.Ar. (*K*) Representative 6‐μm μCT reconstructions of the femur are shown for vehicle‐treated and PTX‐treated mice with trabecular bone on top and cortical bone below. Red scale bar = 500 μm. Mean ± SEM. *p < 0.05; **p < 0.01, ****p < 0.001, or *p* = ns (not significant) by Student's *t* test or Mann‐Whitney test (Tb.N, Ct.Th, and Ct.Ar) following assessment for normal distribution by Shapiro‐Wilk normality test. *n* = 11 mice/group. One femur in the vehicle group was damaged and could not be analyzed. BV = bone volume; BV/TV = trabecular bone volume fraction; Conn.D = connectivity density; Ct.Ar = cortical area; Ct.Th = cortical thickness; CTx = C‐terminal telopeptide of collagen type I; P1NP = N‐terminal propeptide of type‐I procollagen; Tb.N = trabecular number; Tb.Sp = trabecular separation; Tb.Th = trabecular thickness; TV = tissue volume; TV.D = volumetric bone density.

**Fig. 3:**
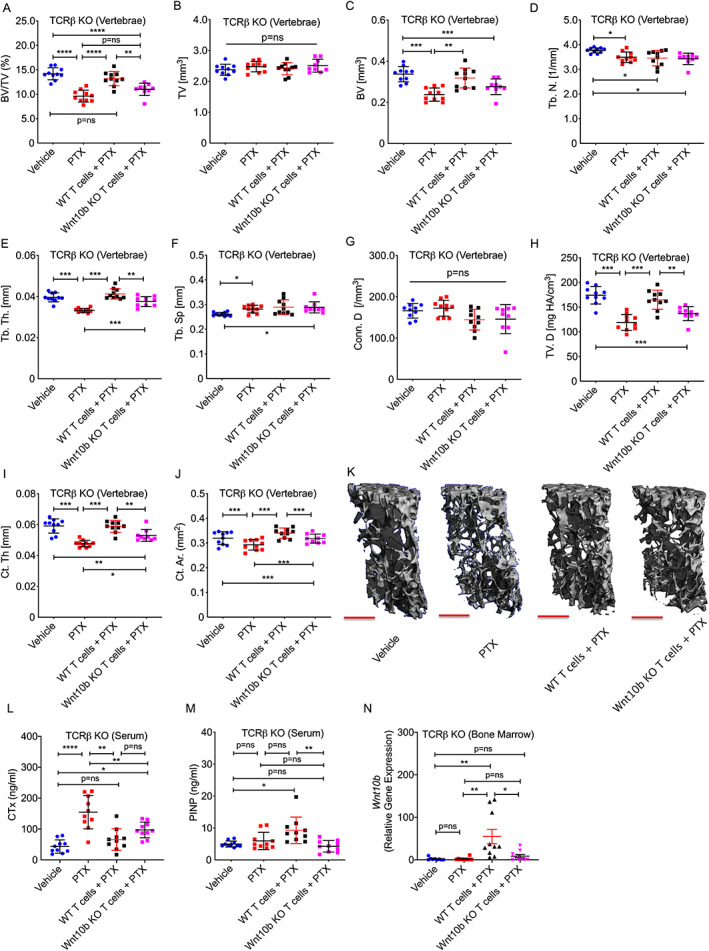
PTX induces vertebral bone loss due to increased resorption, but not formation, in T cell–deficient mice, but is rescued by reconstitution of CD8^+^ T cells from WT, but not Wnt‐10b KO mice. C57BL6/J female TCRβ KO T cell–deficient mice, and TCRβ KO mice reconstituted with WT or CD8^+^ Wnt‐10b KO T cells, were treated with PTX or vehicle (PBS) 5 times/week, for 12 weeks. Vertebral structural indices were analyzed by μCT for: (*A*) BV/TV; (*B*) TV; (*C*) BV; (*D*) Tb.N; (*E*) Tb.Th; (*F*) Tb.Sp; (*G*) Conn.D; and (*H*) TV.D, and for cortical indices: (*I*) Ct.Th and (*J*) Ct.Ar. (*K*) Representative 6‐μm μCT reconstructions. Red scale bar = 500 μm. (*L*) Bone resorption marker C‐terminal telopeptide of CTx and (*M*) bone formation marker P1NP. (*N*) RT‐PCR analysis of *Wnt‐10b* expression in bone marrow. Mean ± SEM. *p < 0.05; **p < 0.01, ***p < 0.001, ****p < 0.001, or *p* = ns (not significant) by one‐way ANOVA or Kruskal‐Wallis test (BV/TV, Tb.N, Tb.Sp, Conn.D, TV.D, and Ct.Th). *n* = 10 mice/group. One extreme outlier in the Wnt‐10b KO T cells + PTX group, with an exceptionally high BV/TV (=17%) and other parameters was excluded from the μCT data. BV = bone volume; BV/TV = trabecular bone volume fraction; Conn.D = connectivity density; Ct.Ar = cortical area; Ct.Th = cortical thickness; CTx = C‐terminal telopeptide of collagen type I; P1NP = N‐terminal propeptide of type‐I procollagen; Tb.N = trabecular number; Tb.Sp = trabecular separation; Tb.Th = trabecular thickness; TV = tissue volume; TV.D = volumetric bone density.

**Fig. 4:**
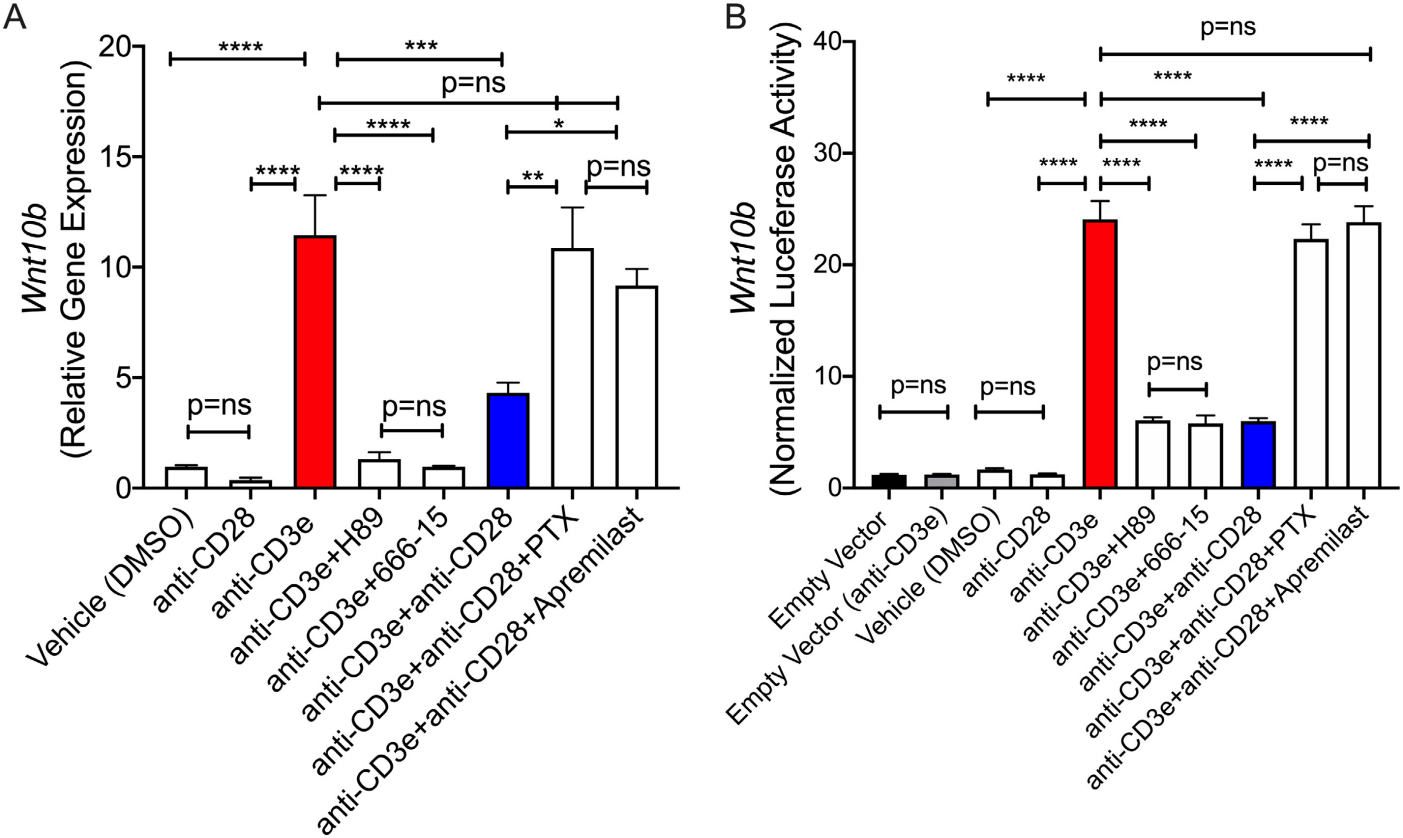
In vitro analysis of the role of the cAMP pathway in *Wnt‐10b* expression by CD8^+^ T cells. The effect of cAMP pathway activators and inhibitors on *Wnt‐10b* gene expression in purified CD8^+^ T cells was assessed by (*A*) RT‐PCR and (*B*) by luciferase assay using a transfected Wnt‐10b promoter reporter and normalized to Renilla. In both (*A*) and (*B*) purified T cells were stimulated with plate bound anti‐CD3e (10 μg/mL) and/or soluble anti‐CD28 activating antibodies (25 μg/mL). Cells were treated with the inducers of cAMP accumulation (PTX [100μM] and apremilast [10μM]) or with H89 (25μM) an inhibitor of PKA or 666‐15 (1μM) a CREB inhibitor. After 6 hours (*A*) mRNA was extracted for RT‐PCR quantification of *Wnt‐10b* expression with normalization to 18S, or (*B*) quantification of luciferase activity using a dual‐luciferase reporter assay with normalization for Renilla expression. Mean ± SEM. *p < 0.05; **p < 0.01, ***p < 0.001, ****p < 0.001, or *p* = ns (not significant) by one‐way ANOVA following assessment for normal distribution by Shapiro‐Wilk normality test. *n* = 4 replicates/group and the data are representative of two independent experiments.

Female C57BL6/J WT mice and T cell receptor β gene (*Tcrb*) KO (TCRβ KO) mice lacking αβ T cells (B6.129P2‐Tcrbtm1Mom/J) were purchased from the Jackson Laboratory (Bar Harbor, ME, USA). *Wnt‐10b* gene KO mice (Wnt‐10b KO) on the C57BL6 background were obtained from a breeding colony maintained at Emory University and were originally gifted by Dr. T.F. Lane (UCLA).^(^ ^12^, ^19^, ^35^ ^)^ We used young mice (8 weeks of age) to maintain consistency with our previous studies of T cell–induced bone anabolism and because both young and skeletally mature mice respond to anabolic stimuli, but younger mice have greater statistic power to detect changes due to increased magnitude of responses.^(^ ^18^ ^)^ We used *n* = 10–11 mice/group based on our previous publications of CD8^+^ T cell anabolism where *n* = 7–8 mice/group^(^ ^18^, ^20^ ^)^ generated significant outcomes. Analyses were performed blinded to the nature of the samples. Animals were distributed into groups based on baseline total body bone mineral density (BMD) quantified by dual‐energy X‐ray absorptiometry (DXA) (PixiMus; GE Medical Systems, Milwaukee, WI, USA) to minimize variance between groups that could bias outcomes in cross‐sectional endpoints. Group baseline total body BMD values (mean ± SD, g/cm^2^) are as follows: Figs. 1 and 2: WT + vehicle (0.0426 ± 0.0023); WT + PTX (0.0425 ± 0.0018). Figures 3 and 4: TCRβ KO + vehicle (0.0459 ± 0.0015); TCRβ + PTX (0.0458 ± 0.0009); TCRβ KO + WT CD8^+^ T cells + PTX (0.0457 ± 0.0011) and TCRβ KO + Wnt‐10b CD8^+^ T cells + PTX (0.0465 ± 0.0017). Percentage change between all groups within an experiment was less than 2%.

### PTX administration to mice

Mice were injected daily (Monday to Friday) for 12 weeks with PTX (50 mg/kg/d) by intraperitoneal injection (i.p.). Control mice were injected with vehicle (phosphate‐buffered saline [PBS]). We elected to use a pharmacological dose used in humans to treat claudication (400 mg/d), which we allometrically scaled (3/4 power scaling) for mouse metabolic equivalency^(^ ^36^ ^)^ to ~50 mg/kg/d.

### T cell adoptive transfer

To test the specific role of Wnt‐10b from CD8^+^ T cells in the response to PTX we generated chimeric mice unable to express Wnt‐10b from their CD8^+^ T cells, but with all other sources of Wnt‐10b intact, as described.^(^ ^12^, ^37^, ^38^, ^39^ ^)^ Briefly, WT or Wnt‐10b KO CD8^+^ T cells were isolated from the spleens of 6‐week‐old to 8‐week‐old female mice using EasySep negative immunomagnetic purification (StemCell Technologies Inc., Kent, WA, USA). Purified CD8^+^ T cells (2 × 10^6^/mouse) were adoptively transferred by tail vein injection into female C57BL6/J TCRβ KO mice at 8 weeks of age. Sham mice were tail vein injected with vehicle (PBS).

### Real‐time RT‐PCR

Whole bone marrow was purified from a single tibia and the pelvis and *Wnt‐10b* expression analysis was performed as described^(^ ^18^ ^)^ on an ABI Prism 7000 instrument (Applied Biosystems, Foster City, CA, USA) using Applied Biosystems master mix and primer sets and probes for murine *Wnt‐10b* (Mm00442104). Fold changes were calculated using the 2^−∆∆Ct^ method^(^ ^22^ ^)^ with normalization to *β‐actin* (Mm 00607939). To quantify the effect of cAMP pathway activators and inhibitors on Wnt‐10b, RT‐PCR was performed as described^(^ ^40^ ^)^ using SYBR green Master Mix (Applied Biosystems) and *Wnt‐10b* mRNA normalized to 18S rRNA, using previously validated primers.^(^ ^40^ ^)^

### Wnt‐10b‐promoter reporter assays

Wnt‐10b‐promoter luciferase assays were performed as described.^(^ ^40^ ^)^ A plasmid containing a −705 to +216 bp DNA sequence of the mouse Wnt‐10b‐promoter was kindly provided by Dr. D. J. Klemm (University of Colorado).^(^ ^27^ ^)^ CD8^+^ T cells were purified from disaggregated spleen by negative selection EasySep Mouse CD8^+^ T cell Isolation Kit (StemCell Technologies Inc.) and transfected using an Amaxa Mouse T Cell Nucleofector Kit (Lonza, Basel, Switzerland) with 3.6 μg of empty vector (pGL3‐basic) or Wnt‐10b‐reporter construct and 0.4 μg TK‐pRL Renilla transfection control vector. Twenty‐four hours after transfection cells were stimulated with plate‐bound anti‐CD3e (10 μg/mL) and soluble anti‐CD28 activating antibodies (25 μg/mL) from BioLegend (San Diego, CA, USA). PTX (100μM) and other activators and inhibitors of the cAMP/PKA/CREB pathway included: H89 a PKA inhibitor (25μM in dimethylsulfoxide [DMSO]); 666‐15 (1μM in DMSO) a CREB inhibitor, and the PDE inhibitor PTX (100μM in PBS), and apremilast (10μM in DMSO) a PDE4 inhibitor, all from R&D Systems, Minneapolis, MN, USA). After 6 hours, luciferase activity was quantified using a dual‐luciferase reporter assay kit (Promega BioSciences, San Luis Obispo, CA, USA) on a Turner BioSystems Inc. (Sunnyvale, CA, USA) luminometer and data normalized for Renilla expression.

### Micro‐computed tomography

Micro‐computed tomography (μCT) was performed in L_3_ vertebrae and the distal femoral metaphysis using a μCT40 scanner (Scanco Medical AG, Brüttisellen, Switzerland) as described.^(^ ^18^, ^20^ ^)^ We scanned 405 tomographic slices at a voxel size of 6 μm (70 kVp and 114 mA, and with 200 ms integration time) at the L_3_ vertebra (total length of 2.4 mm) and 100 tomographic slices at the distal femoral metaphysis. Trabecular bone was segmented from the cortical shell for a total length of 0.6 mm beginning 0.5 mm from the distal growth plate. Cortical bone was quantified at the femoral mid‐diaphysis from 100 tomographic slices (total length of 0.6 mm). We used the thresholding approach described by Bouxsein and colleagues,^(^ ^41^ ^)^ which is recommended by the μCT manufacturer, and involves a visual inspection and comparison of preview and slice wise grayscale two‐dimensional (2D) images. The same threshold value was used for all measurements.

### Quantitative bone Histomorphometry

Bone histomorphometry was performed by the Center for Metabolic Bone Disease‐Histomorphometry and Molecular Analysis Core Laboratory of the University of Alabama at Birmingham, on trichrome‐stained plastic‐embedded sections of calcein labeled femurs from vehicle and PTX‐injected mice. For dynamic bone formation indices mice were injected subcutaneously with calcein at day 10 and day 3 before euthanasia.

### Biochemical indices of bone turnover

Serum biochemical markers of bone resorption (C‐terminal telopeptide of collagen [CTx]) and of bone formation (N‐terminal propeptide of type I procollagen [P1NP]) were quantified in mice serum using ELISAs from Immunodiagnostic Systems Inc. (Gaithersburg, MD, USA).

#### Statistical analysis

Statistical significance was determined using Prizm version 8.4.3 for Macintosh (GraphPad Software Inc., La Jolla, CA, USA). Gaussian distribution was assessed by Shapiro‐Wilk test. Parametric two sample comparisons were made using unpaired two‐tailed Student's *t* test. Welch's correction was applied to samples with unequal variance based on *F*‐test. For nonparametric data Mann‐Whitney test was used. Multigroup comparisons (≥3) were made using one‐way ANOVA with Tukey‐Kramer post hoc test for parametric data, or Kruskal‐Wallis test with Dunn's post hoc test, for nonparametric data. Outliers were assessed by Grubb's test with α = 0.05. All available data were used for analysis unless otherwise stated in the figure legend. Graphical data are presented as mean ± standard error of the mean (SEM) with dots representing individual mice; *p* < 0.05 was considered statistically significant.

## Results

### Treatment of WT mice with PTX increases trabecular and cortical parameters of bone mass in the lumbar spine

To investigate the net effect of cAMP elevation in vivo, we treated healthy WT female C57BL6/J mice, 8 weeks of age, with PTX, a potent broad‐spectrum PDE inhibitor, or with vehicle (PBS). Mice were injected i.p. five times/week, for 12 weeks and L_3_ lumbar vertebrae were analyzed by μCT to obtain separate high‐resolution microarchitectural analyses of the trabecular and cortical compartments.

Trabecular bone volume fraction (BV/TV) a key index of trabecular bone mass, was significantly increased in PTX‐treated WT mice (Fig. 1*A*). This was a consequence of increased bone volume (BV) rather than a change in tissue volume (TV), an index reflecting changes in trabecular compartment size (Fig. 1*B*,*C*). The structural indices trabecular number (Tb.N) and trabecular thickness (Tb.Th) were both significantly increased by PTX treatment (Fig. 1*D*,*E*), consistent with a corresponding decline in trabecular separation (Tb.Sp) (Fig. 1*F*). Connectivity density (Conn.D) and volumetric bone density (TV.D) measurements were also significantly increased (Fig. 1*G*,*H*).

Two key indices of cortical mass: cortical area (Ct.Ar) and cortical thickness (Ct.Th), were significantly increased by PTX treatment (Fig. 1*I*,*J*). Representative μCT reconstructions of L_3_ vertebrae are shown for vehicle‐treated and PTX‐treated WT mice (Fig. 1*K*).

#### Biochemical indices of bone formation and resorption are increased in PTX‐treated WT mice

To assess PTX‐induced bone turnover we examined changes in bone resorption and bone formation by quantifying serum biochemical markers of bone resorption (CTx) and of bone formation (P1NP). Both CTx (Fig. 1*L*) and P1NP (Fig. 1*M*) were significantly increased in PTX‐treated WT mice. The data suggest a high rate of bone turnover (increased formation and resorption) but with net gain in BV/TV in the axial skeleton (Fig. 1*A*).

#### *Wnt‐10b* expression in the bone marrow is increased in PTX‐treated WT mice

We have previously reported that T cells activated in the absence of CD28 co‐stimulation in vitro express *Wnt‐10b*, which could account for the bone anabolic activity of abatacept in mice.^(^ ^18^, ^19^ ^)^ To investigate whether PTX promotes *Wnt‐10b* expression, we performed RT‐PCR in whole bone marrow from vehicle‐treated and PTX‐treated WT mice. The data reveal a significant increase in *Wnt‐10b* expression in PTX‐treated mice (Fig. 1*N*).

### Treatment of healthy WT mice with PTX fails to increase trabecular and cortical parameters of bone mass in the femur

In contrast to the vertebrae, BV/TV in the femur was not significantly increased by PTX (Fig. 2*A*), although interestingly, Tb.N was significantly increased whereas Tb.Th was significantly diminished (Fig. 2*D*,*E*). Tb.Sp was modestly decreased (Fig. 2*F*), whereas TV and BV (Fig. 2*B*,*C*) and Conn.D and TV.D (Fig. 2*G*,*H*) were unchanged. In the cortical compartment, Ct.Th was significantly diminished but Ct.Ar remained unchanged (Fig. 2*I*,*J*).

Representative μCT reconstructions of femoral cortical and trabecular bone are shown for vehicle‐treated and PTX‐treated WT mice (Fig. 2*K*).

#### Analysis of PTX‐treated WT femurs by histomorphometry

Histomorphometry performed on isolated calcein labeled femurs from vehicle‐treated and PTX‐treated WT mice revealed no significant changes in osteoclast surface normalized for bone surface (Oc.S/BS); however, the number of osteoclasts (N.Oc/BS) were significantly increased (Table 1).

**Table 1:** Histomorphometry of Vehicle‐Treated and PTX‐Treated Mice (Femur)

Parameters	Vehicle‐treated (mean ± SD)	PTX‐treated (mean ± SD)	Percent change	*p*
Static indices				
Oc.S/BS	5.243 ± 2.115	6.332 ± 2.297	20.8	0.1043
N.Oc/BS	1.815 ± 0.623	2.243 ± 0.706	23.6	0.0315
Ob.S/BS	40.478 ± 9.955	42.485 ± 10.137	5.0	0.4603
N.Ob/BS	30.157 ± 5.807	30.885 ± 6.737	2.4	0.6719
Dynamic indices				
MAR (μm/d)	1.272 ± 0.192	1.304 ± 0.276	2.5	0.6608
BFR/BS (μm/d)	0.524 ± 0.082	0.458 ± 0.118	−12.6	0.0607

Values of *p* represent Student's *t* tests. *n* = 11 mice/group. Percent change is the percentage change between the vehicle‐treated and PTX‐treated group.

BFR = bone formation rate; BS = bone surface; MAR = mineral apposition rate; N.Ob = osteoblast number; N.Oc = osteoclast number; Ob.S = osteoblast surface; Oc.S = osteoclast surface.

Neither the static indices osteoblast surface (Ob.S/BS) and the number of osteoblasts (N.Ob/BS), nor the dynamic indices mineral apposition rate (MAR) and bone formation rate (BFR/BS) were significantly changed by PTX treatment in the femur (Table 1), consistent with a lack of BV/TV increase at this site.

#### PTX induces bone loss in T cell–deficient TCRβ KO mice, but bone loss is rescued by reconstitution of TCRβ KO mice with CD8 ^+^ T cells from WT, but not by reconstitution of TCRβ KO mice with CD8 ^+^ T cells from Wnt‐10b KO mice

To further explore the role of CD8^+^ T cells, and their Wnt‐10b production, in the bone anabolic and catabolic effects of PTX, we administered PTX to TCRβ KO mice lacking αβ T cells, the TCR expressed by the vast majority of T cells in the body. To demonstrate the specific role of Wnt‐10b production from CD8^+^ T cells in the bone anabolic effect of PTX we also reconstituted, by adoptive transfer, TCRβ KO mice with CD8^+^ T cells derived from *Wnt‐10b* KO mice. Control mice were reconstituted with WT CD8^+^ T cells.

Using μCT to assess changes in vertebral bone mass, we found that PTX not only failed to promote trabecular bone accretion in the TCRβ KO mice, but BV/TV, BV, Tb.N, Tb.Th, and TV.D were all significantly diminished by PTX treatment (Fig. 3*A*,*C*–*E*). Consistent with the decline in BV/TV, Tb.Sp was increased (Fig. 3*F*), while TV and Conn.D were not significantly affected by PTX treatment (Fig. 3*B*,*G*).

Adoptive transfer of WT CD8^+^ T cells into TCRβ KO mice rescued mice from trabecular bone loss; however, TCRβ KO mice transplanted with T cells lacking expression of Wnt‐10b, were not protected from bone loss (Fig. 3*A*,*C*,*D*,*H*).

Similar to the trabecular compartment, the key cortical indices Ct.Th and Ct.Ar were significantly decreased by PTX treatment in mice lacking CD8^+^ T cells (Fig. 3*I*,*J*) and WT CD8^+^ T cells reversed these changes. By contrast, transplantation of Wnt‐10b KO CD8^+^ T cells into TCRβ KO mice only partly reversed the changes in Ct.Th and Ct.Ar (Fig. 3*J*). Representative μCT reconstructions of L_3_ vertebrae are shown for each group (Fig. 3*K*).

#### Biochemical indices of bone resorption, but not bone formation, are increased by PTX treatment of T cell–deficient TCRβ KO mice

The biochemical marker of bone resorption CTx, reflected enhanced bone resorption induced by PTX in T cell–deficient mice and was significantly reversed by transplantation of WT CD8^+^ T cells and partly reversed by Wnt‐10b KO CD8^+^ T cells (Fig. 3*L*). Bone formation marker P1NP was not increased by PTX treatment of TCRβ KO mice, but was rescued by transplantation of WT, but not Wnt‐10b KO CD8^+^ T cells (Fig. 3*M*).

#### *Wnt‐10b* expression in the bone marrow is not increased by PTX treatment of TCRβ KO mice, but is rescued by reconstitution of WT, but not Wnt‐10b KO, CD8 ^+^ T cells

Consistent with the hypothesis that CD8^+^ T cells are the relevant source of PTX‐induced Wnt‐10b, *Wnt‐10b* expression in the bone marrow was not increased in PTX‐treated TCRβ KO mice, but was rescued by transplantation of TCRβ KO mice with WT, but not Wnt‐10b KO CD8^+^ T cells (Fig. 3*N*).

Taken together, these data suggest that the bone anabolic effects (increased P1NP and vertebral BV/TV increase) induced by PTX are mediated through CD8^+^ T cells and specifically through Wnt‐10b production by CD8^+^ T cells. By contrast, T cells are dispensable for the bone catabolic action (increased CTx in WT and TCRβ KO mice, and BV/TV loss in TCRβ KO mice) induced by PTX, and counteract in part, the magnitude of the resorptive activity of PTX.

#### *Wnt‐10b* gene expression is upregulated in WT CD8 ^+^ T cells activated in the absence of CD28 co‐stimulation and in dual CD3‐activated and CD28‐activated CD8 ^+^ T cells treated with PDE inhibitors, whereas inhibitors of PKA and CREB suppress *Wnt‐10b* expression

To investigate the molecular signals that drive *Wnt‐10b* expression in CD8^+^ T cells, we treated purified primary WT CD8^+^ T cells with inhibitors and activators of the cAMP pathway and quantified *Wnt‐10b* gene expression by RT‐PCR. Activation of T cells by CD3 activation (using an anti‐CD3e activating antibody) in the absence of CD28 co‐stimulation led to significant induction of *Wnt‐10b* gene expression (Fig. 4*A*; red bar). As expected, *Wnt‐10b* expression was significantly diminished by CD28 co‐stimulation in CD3‐activated T cells (Fig. 4*A*; blue bar). Suppression of cAMP degradation using the broad‐spectrum PDE inhibitor PTX, or the PDE4‐specific inhibitor apremilast, both reversed the suppressive effect of dual CD3 and CD28‐induced T cell activation, on *Wnt‐10b* gene expression. CD3‐induced *Wnt‐10b* expression was significantly suppressed by the PKA inhibitor H89 and by the CREB activation inhibitor 666‐15 (Fig. 4*A*), attesting to a role for these downstream cAMP‐signal transduction mediators.

Finally, we interrogated *Wnt‐10b* gene activation in WT CD8^+^ T cells using a Wnt‐10b promoter‐luciferase reporter construct that was transfected into purified primary CD8^+^ T cells and treated with activators and inhibitors of the cAMP pathway, as described above for the RT‐PCR study, following activation in the presence or absence of CD28 co‐stimulation. This study produced almost identical outcomes to those reported above for mRNA quantification (Fig. 4*B*) and support a direct role of the cAMP pathway in Wnt‐10b promoter activation.

## Discussion

The cAMP signal transduction pathway is recognized as a key suppressor of T cell activation,^(^ ^22^ ^)^ and promotes a state recognized to stimulate Wnt‐10b secretion and bone anabolism.^(^ ^18^ ^)^ In this study, we examined the cellular and molecular mechanisms by which the pharmacological PDE inhibitor PTX drives bone anabolic activity. Our data confirm the bone anabolic potential of PDE inhibition and demonstrate a previously unrecognized requirement for CD8^+^ T cells, and for Wnt‐10b production, in PTX‐induced bone formation. A key role for CD8^+^ T cells was evidenced by the loss of bone anabolic activity in T cell KO mice and restoration of bone formation by transplantation of WT CD8^+^ T cells. By contrast, reconstitution by CD8^+^ T cells lacking a functional *Wnt‐10b* gene, failed to restore bone formation, corroborating a specific role for this bone anabolic Wnt ligand in PTX‐induced bone anabolism. In vitro molecular signaling studies further demonstrate that activation of the cAMP signal transduction pathway in CD8^+^ T cells drives *Wnt‐10b* transcription through classical downstream cAMP mediators including PKA and CREB.

Administration of PTX to WT mice resulted in a high bone turnover state characterized by accelerated bone formation and bone resorption, which competed against each other. Over an extended period of 3 months, the balance of bone resorption and formation was in favor of trabecular and cortical bone volume gain in the axial skeleton. By contrast, we did not observe significant bone acquisition in the femur and although PTX appeared to promote an increase in femoral Tb.N, this was offset by a simultaneous decline in Tb.Th. The opposing actions of formation and resorption may have led to an expansion of new trabecular bone elements through increased bone formation, but with denuding of existing element thickness through competing bone resorption. An alternative explanation for increased Tb.N, in the face of diminished Tb.Th, is that elevated bone resorption can lead to the perforation of trabecular rods, leading to an apparent increase in rod number. This latter explanation is consistent with our histomorphometry which revealed a significant increase in the N.Oc/BS while the N.Ob/BS was not significantly increased, suggesting that the dominant activity in the femur at the time of mice euthanasia was pro‐resorptive, rather than anabolic.

In the absence of T cells (TCRβ KO mice), the net effect of PTX was bone loss, the result of induced bone resorption in the face of a stagnant anabolic response. As loss of T cells failed to abrogate the pro‐resorptive effects of PTX, the data suggest that T cells are obligatory for PTX‐induced bone anabolism, but are dispensable for PTX‐induced bone resorption. This is consistent with previous studies reporting that PDE inhibitors such as PTX, may upregulate RANKL production by osteoblasts.^(^ ^42^ ^)^

Unlike previous studies using PTX in intact WT animals^(^ ^28^ ^)^ that were found to be bone anabolic in the femur, the balance of formation and resorption in our study led to significant bone gain in the vertebrae only. The reason for this is unclear, but interestingly, we previously observed a similar predilection for bone gain in the vertebrae when we treated mice with an antibody that inhibits CD40 ligand co‐stimulation.^(^ ^20^ ^)^ Furthermore, clinical studies have revealed that the axial skeleton undergoes a much more robust response to teriparatide than the appendicular skeleton.^(^ ^43^ ^)^

One explanation for this is that the size of the CD8^+^ T cell pool in C57BL6 mice is almost 60% greater in the vertebrae that in femoral bone marrow.^(^ ^44^ ^)^ This expanded number of CD8^+^ T cells in the vertebrae may have been sufficient to sustain an anabolic response, while the lower total number of CD8^+^ T cells in the femur may have been inadequate to meet the threshold concentration of Wnt‐10b required to promote sufficient bone formation to overcome the direct effects of PTX on osteoclastic bone resorption.

Another difference is that the previous study used a higher dose of PTX (100–200 mg/kg),^(^ ^28^ ^)^ while our study used 50 mg/kg/d, a dose allometrically scaled to mouse equivalency from the clinical dose used to ameliorate claudication in humans. We chose this lower dose because, in a pilot study performed in preparation for our experiments, we found that PTX doses >50 mg/kg caused the mice to display symptoms of illness, such as lethargy. Indeed, systemic global PDE inhibitors, such as PTX and rolipram, are used sparingly in humans because they are associated with similar side effects. Although we did not observe obvious signs of lethargy in our study, it is possible that animal activity may have been diminished, causing reduced biomechanical loading of the femurs, relative to control mice that may have favored resorption over formation at this site.

Although Wnt‐10b expression in bone marrow was significantly elevated in response to PTX (Fig. 1*N*), there was no significant difference in BV/TV and bone formation by histomorphometry, in the femur. A likely explanation for this is that bone marrow for RT‐PCR studies was derived predominantly from the marrow‐rich pelvis and this bone marrow may more closely represent vertebral bone marrow than femoral bone marrow, in terms of the numbers and/or distribution/localization of CD8^+^ T cells.

Another apparent discrepancy is that the bone turnover markers suggest that both formation and resorption were promoted by PTX, whereas the histomorphometry only revealed increases in the N.Oc/BS. The likely explanation is that as evidenced by the μCT data, bone formation in the femur was muted and did not lead to a significant increase in bone volume. Another possible explanation for osteoblast numbers not being increased is that preexisting osteoblasts may have been transiently activated, rather than de novo formation of new osteoblasts. This may have been sufficient to offset a slight increase in bone resorption, but inadequate to promote an increase in volume.

Although reconstitution of WT CD8^+^ T cells rescued PTX‐induced bone loss and restored bone formation, BV/TV was not significantly increased in the vertebrae relative to untreated vehicle controls. This is likely a limitation of the adoptive transfer technique in which the number and distribution of reconstituted T cells do not reach WT levels due to contraction of the T cell niche in immunodeficient animals and hence available space to accommodate T cells is diminished.^(^ ^45^ ^)^ The anabolic response may thus have been sufficient to blunt bone loss, but inadequate to promote excess bone gain.

As with PTX, PTH also promotes bone formation in part, by eliciting Wnt‐10b production by T cells, although the signaling mechanisms involved are indirect and act through promotion of regulatory T cells (Tregs), which lead to the rebalancing of nuclear factor of activated T‐cells (NFAT) and suppressor of mothers against decapentaplegic (SMAD) transcription factors at the *Wnt‐10b* promoter.^(^ ^11^ ^)^ In the case of PTX, cAMP induces PKA. leading to activation of CREB, driving Wnt‐10b promoter activity. This was evidenced by pharmacological ablation of PKA and CREB in reporter assays which abrogated cAMP‐induced Wnt‐10b expression and Wnt‐10b promoter activation.

**Fig. 5:**
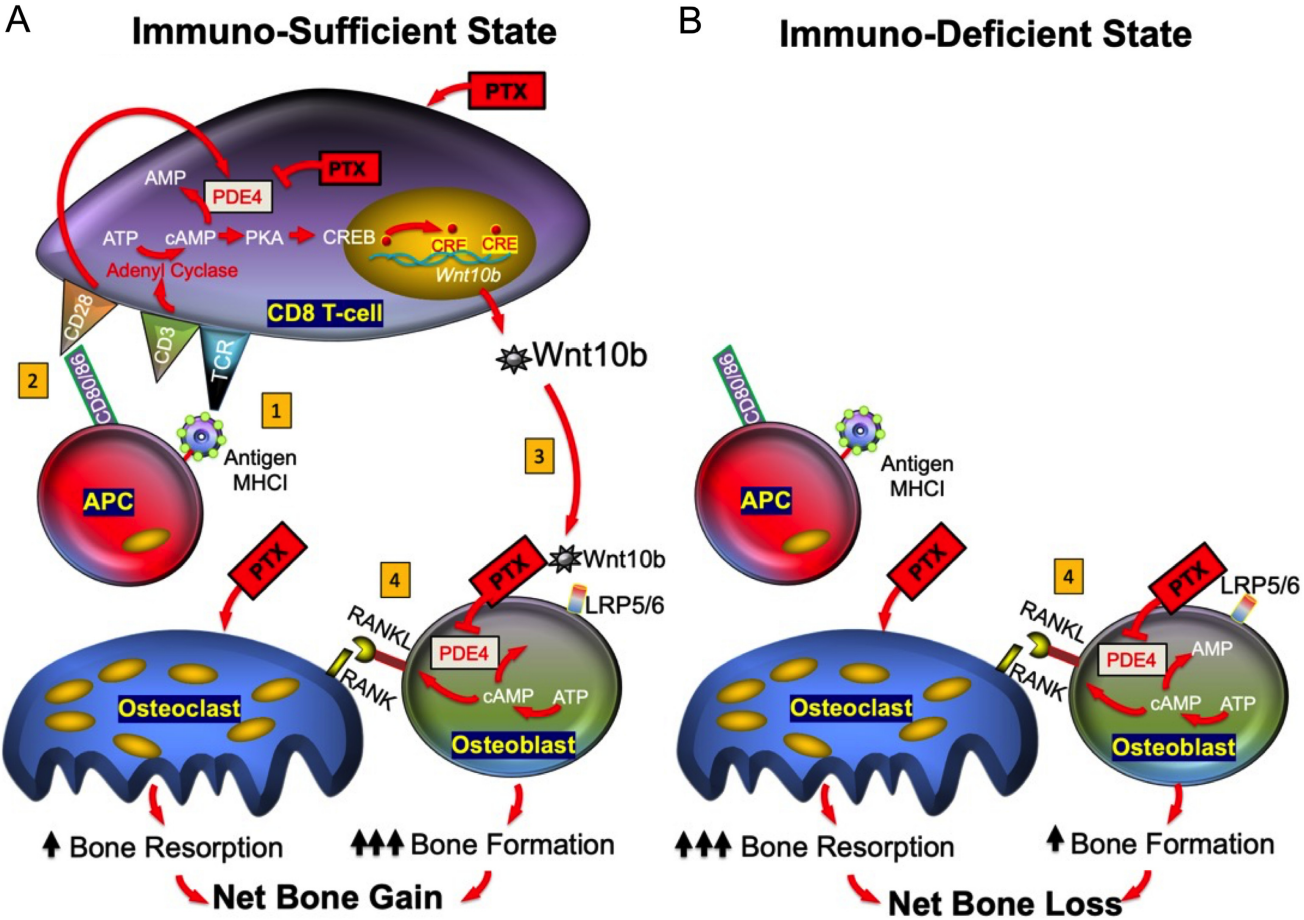
Model for PDE‐induced bone anabolism by CD8^+^ T cells. (*A*) Under immunosufficient conditions, (1) APCs present antigen to the TCR activating the associated CD3 complex (signal 1) and leading to activation of adenylate cyclase with synthesis of cAMP. (2) APC‐expressed CD80/CD86 ligands activate T cell CD28 receptors (signal 2) promoting catabolism of cAMP to signaling inert AMP, through PDE4. PDE inhibitors such as PTX, counteract cAMP degradation allowing accumulation of cAMP. cAMP activates PKA, which in turn activates CREB. CREB translocates to the nucleus and associates with CREs in gene promoters, inducing transcription of target genes including *Wnt‐10b*. (3) Wnt‐10b protein binds to LRP5/6 receptors leading to osteoblastogenesis/osteoblast activation and driving bone formation. (4) Direct effects of PDE‐inhibitor on osteoclasts and/or osteoblasts, induce resorption, causing a high bone turnover state, but with net gain of bone mass in the axial skeleton. (*B*) Under immunodeficient conditions the catabolic effects of PDE dominate, leading to bone loss. APC = antigen presenting cell; CRE = CREB responsive element; CREB = cAMP responsive element binding protein; LRP5/6 = low‐density lipoprotein receptor‐related protein 5/6; PDE4 = phosphodiesterase‐4; PKA = protein kinase A; TCR = T cell receptor.

Based on our data, we propose a model (Fig. 5) to explain the bone anabolic actions of the PDE inhibitor PTX. In the immunosufficient state (Fig. 5*A*), APC bearing antigen activates the TCR and the associated CD3 complex. CD3 activation leads to induction of adenylate cyclase causing synthesis of cAMP from adenosine triphosphate (ATP). Co‐stimulation through CD28 activates PDE4, promoting catabolism of the active signaling molecule cAMP, to the signaling inert AMP, driving T cell activation and effector function. Blocking cAMP catabolism by inhibiting PDE with PTX counteracts PDE‐induced cAMP removal and promotes accumulation of cAMP. cAMP stimulates activation of PKA and phosphorylation and activation of CREB. CREB translocates to the nucleus and binds to CRE within target genes involved in T cell anergy including *Il‐2*, but also of *Wnt‐10b*. Wnt‐10b secreted by CD8^+^ T cells binds to low‐density lipoprotein receptor‐related protein 5/6 (LRP5/6) receptors on bone marrow stromal cells (BMSCs) and/or osteoblasts, leading to osteoblastic differentiation and/or activation of existing osteoblasts, causing bone synthesis. The direct effect of PTX on osteoblasts may lead to expression of RANKL that drives a competing bone catabolic event, causing an increase in bone resorption and development of a high bone turnover state. By contrast, under immunodeficient conditions (Fig. 5*B*) the direct catabolic effects of PDE on bone cells dominate, leading to bone loss.

Although TCRβ KO mice are an experimental animal model, immunocompromised human populations do exist, including HIV‐infected subjects and patients receiving chemotherapeutic drugs, and subjects receiving immunosuppressive therapies to counteract transplant rejection, or for other inflammatory diseases, may be at risk of bone loss associated with PDE‐based therapies.

In conclusion, our studies demonstrate that CD8^+^ T cells are required for PDE‐induced bone anabolism and involve activation of *Wnt‐10b* gene transcription, induced by the cAMP signal transduction pathway.

Selective targeting of CD8^+^ T cells with PDE inhibitors, to maximally promote bone anabolism, while minimizing the stimulatory effects of PDE inhibition on bone resorption, may constitute a novel therapeutic approach to rejuvenate bone and delay fracture in multiple osteoporotic conditions.

## Author contributions

**Susanne Roser‐Page:** Formal analysis; investigation; methodology; writing – review and editing. **Daiana Weiss:** Formal analysis; investigation; methodology; writing – review and editing. **Tatyana Vikulina:** Formal analysis; investigation; methodology; writing – review and editing. **Mingcan Yu:** Formal analysis; investigation; methodology; writing – review and editing. **Roberto Pacifici:** Resources; writing – review and editing. **M. Neale Weitzmann:** Conceptualization; formal analysis; funding acquisition; project administration; supervision; writing – original draft; writing – review and editing.

## Conflicts of Interest Statement

All authors declare that they have no conflicts of interest.

### Peer review

The peer review history for this article is available at https://publons.com/publon/10.1002/jbm4.10636.

## Data Availability

The data that support the findings of this study are available from the corresponding author upon reasonable request.
